# miRkit: R framework analyzing miRNA PCR array data

**DOI:** 10.1186/s13104-021-05788-1

**Published:** 2021-09-26

**Authors:** Maria Tsagiopoulou, Anastasis Togkousidis, Nikolaos Pechlivanis, Maria Christina Maniou, Aristea Batsali, Angelos Matheakakis, Charalampos Pontikoglou, Fotis Psomopoulos

**Affiliations:** 1 Institute of Applied Biosciences, Centre of Research and Technology Hellas, 57001 Thessaloniki, Greece; 2 Haemopoiesis Research Laboratory, School of Medicine, University of Crete, 71003 Heraklion, Greece; 3 Department of Hematology, School of Medicine, University of Crete, 71003 Heraklion, Greece; 4 Department of Genetics, Development and Molecular Biology, School of Biology, Aristotle University of Thessaloniki, 54124 Thessaloniki, Greece

**Keywords:** miRNA, qPCR, RT-PCR, PCR array, GO, KEGG

## Abstract

**Objective:**

The characterization of microRNAs (miRNA) in recent years is an important advance in the field of gene regulation. To this end, several approaches for miRNA expression analysis and various bioinformatics tools have been developed over the last few years. It is a common practice to analyze miRNA PCR Array data using the commercially available software, mostly due to its convenience and ease-of-use.

**Results:**

In this work we present miRkit, an open source framework written in R, that allows for the comprehensive analysis of RT-PCR data, from the processing of raw data to a functional analysis of the produced results. The main goal of the proposed tool is to provide an assessment of the samples’ quality, perform data normalization by endogenous and exogenous miRNAs, and facilitate differential and functional enrichment analysis. The tool offers fast execution times with low memory usage, and is freely available under a ΜΙΤ license from https://bio.tools/mirkit. Overall, miRkit offers the full analysis from the raw RT-PCR data to functional analysis of targeted genes, and specifically designed to support the popular miScript miRNA PCR Array (Qiagen) technology.

## Introduction

MicroRNAs are small non-coding RNA molecules with a critical role in gene expression regulation [1]. They are implicated in mRNA post-transcriptional modulation in the cell as well as released into circulation and transferred to other target cells [2]. For this reason, and beyond their key role in intracellular pathways [2], miRNAs have emerged as biomarkers in clinical medicine [3] and are thought to represent appealing novel therapeutic modalities [4]. Also, the expression levels of miRNAs are known to be deregulated in diseases and malignancies [5, 6].

Various approaches have been used to profile the expression of miRNAs [4] such as RT-PCR arrays, microarrays, small RNA-seq [7]. Quantitative real-time PCR (RT-PCR) assays are sensitive and specific in detecting and quantifying the expression of miRNAs in the human miRNA genome (miRNome) [4]. Within this context, the commercially available human miRNome miScript miRNA PCR Array (Qiagen) can be used to profile the 1066 most abundantly expressed and best characterized miRNA sequences in the human miRNome, as annotated in miRBase Release 16 (www.miRBase.org).

Raw RT-PCR data are typically analyzed using the manufacturer’s software. In fact, several open-source packages analyze RT-PCR data with the traditional Ct (threshold cycle) quantification approach [8], ignoring more sophisticated and publicly available methods to analyze the expression profiles.

Currently, and going beyond miRNA data, there are plenty of options for differential expression analysis in which the user can employ a larger range of more sophisticated methods, ending up with values for logFC and adj-pvalue. To assess the potential benefits of analyzing this data with an open source tool, we developed miRkit, a framework written in R, specific for miScript miRNA PCR Array (Qiagen) technology. The proposed toolset offers the whole analysis of the raw RT-PCR data completely automated, including quality control of the samples, normalization of endogenous and exogenous controls, differential expression analysis and functional analysis of targeted genes. Finally, the package has fast execution time and uses very low memory.

## Main text

### Implementation

#### Input data

The main R script of the workflow reads the input data from three distinct files stored in a folder:

1. Count table: This is the main data file, containing the different samples on columns and the measurement of each well on the rows. The proposed tool is applicable on miScript miRNA PCR Array (Qiagen) which contains 384 wells and examines 372 miRNAs, 12 controls. Specifically, each well of 372/384 contains a miScript Primer Assay for a miRNome or pathway/disease/functionally-related mature RNA. Moreover, 2 wells contain replicate C. elegans miR-39 miScript Primer Assays and can be used as an alternative normalizer for array data (Ce), 6 wells contain an assay for a different snoRNA/snRNA that can be used as a normalization control for the array data. Finally, there are two wells which contain replicate miRTC Primer Assays (RTC) and two wells that contain positive PCR controls (PPC).
2. Metadata: This file includes a list of sample IDs and the corresponding group e.g. normal/tumor
3. Annotation of miRNAs well: A file that links the information of the well with the examined miRNA.

#### Workflow

**Fig. 1.**
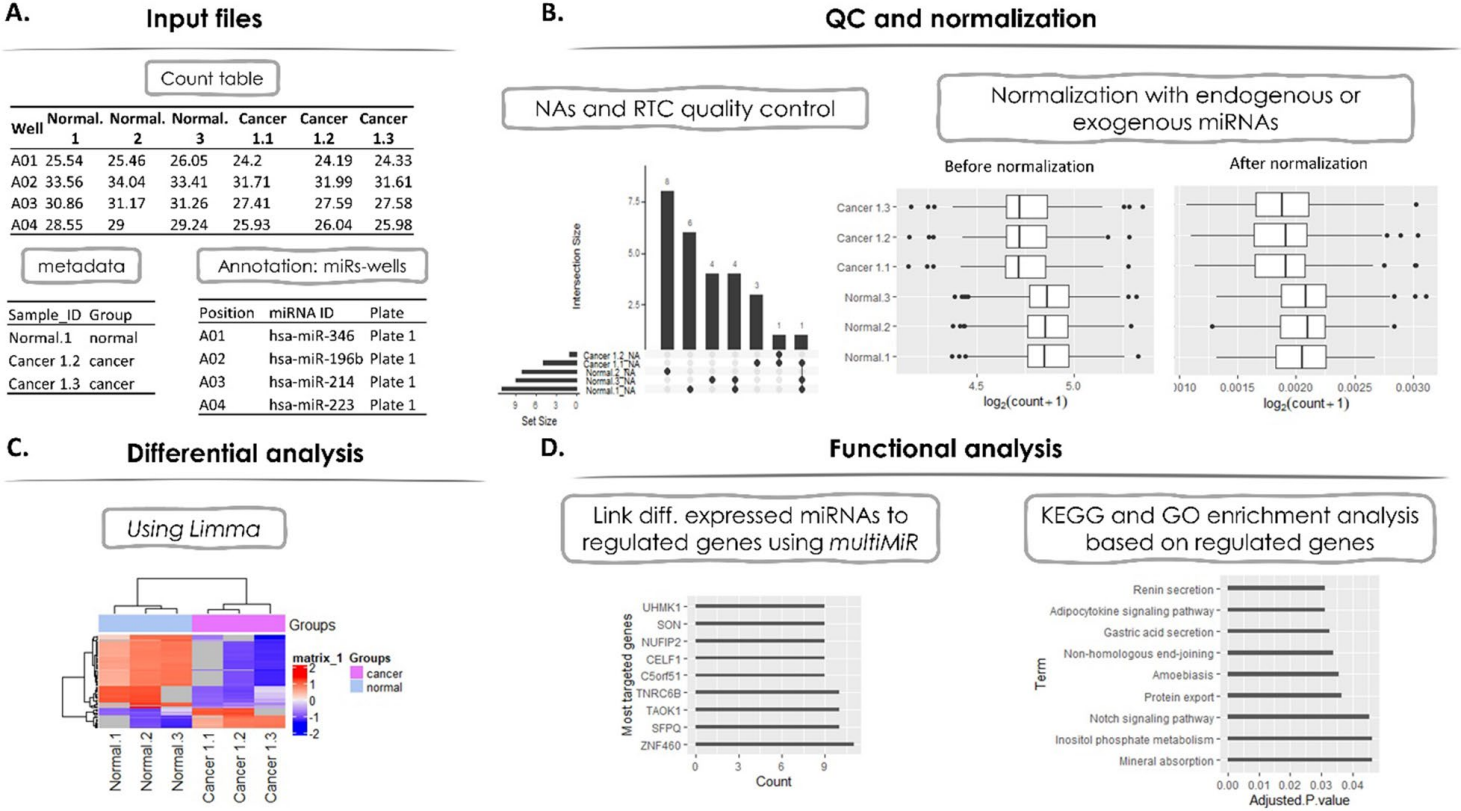
Graphical representation of working cases of the miRkit tool. **A** An example of the input file format to miRkit. **B** Quality control and normalization per sample. **C** Differential expression analysis between normal and cancer miRNAs. **D** Linking to regulated genes and annotating with KEGG and GO data bases

The framework is implemented into three distinct phases, as shown in Fig. 1; [1] QC and normalization, [2] differential analysis and [3] functional analysis. Specifically:

1. QC and normalization.
  The quality control process examines two different aspects:
  - the maximum percentage of not detected or not available values (NA’s) in each column. This quality threshold is defined by the user. If a sample fails the comparison then it is excluded from the rest of the analysis.
  - the ratio between reverse transcription control (RTC) assay, which detects an artificial RNA template, and positive PCR controls (PPC), which monitor for PCR inhibitors, is calculated and a standard threshold is used to validate the reverse transcription efficiency which descripted by Qiagen. Specifically, if the ratio is < 5 the sample is passed this quality step.
  The data normalization module includes the option of endogenous and exogenous miRNA approach. Normalization of miRNAs using the endogenous controls corrects for factors that could influence the quantification such as different quantities of input RNA, RNA degradation, presence of inhibitors, errors in sample handling. Exogenous controls are typically used on difficult samples such as plasma/serum or other biofluids. Many exogenous controls are not present in humans so it is a good exogenous control for human samples. MiScript PCR Controls are primers designed to quantify a panel of 5 snoRNAs (SNORD61, SNORD68, SNORD72, SNORD95, and SNORD96A) and the snRNA RNU6B (RNU6-2) as endogenous controls and cel-miR-39 as exogenous.
  The output of this step is the normalized data matrix that includes the samples which passed the NA’s criterion. Additionally, a visualization option is available, which allows to generate figures that are automatically stored within the analysis folder, and include an upset plot for the NA’s distribution and boxplots with counts before and after the normalization.
2. Differential analysis
  This module is performed using the limma package in R [9]. The output includes the differentially expressed miRNAs using a user-defined adjusted p-value as a threshold. Moreover, a hierarchical cluster analysis is performed at this stage and a corresponding heatmap is constructed and stored in the analysis directory.
3. Functional analysis
  The downstream analysis links the differentially expressed miRNAs with the regulated genes using the multiMIR package, which includes several databases such as mirtarbase [10], tarbase [11], diana_microt [12] etc. for both predicted and vali-dated targets. Moreover, the targeted genes of the differentially expressed miRNAs are used for KEGG and Gene ontology (GO) enrichment analysis, as facilitated by the *enrichR* package [13]. Finally, barplots that present the results of the enrichment analysis are stored in the analysis folder.
  At the end of the entire process miRkit produces a list of tables containing all significant events along with the corresponding plots, separated into different folders. Additionally, a report file is automatically exported. The report contains information of the particular execution process, including the user-defined criteria, the rationale for the excluded samples, the overall time required for execution and the total memory usage.

### Case study

The miRkit was applied on artificial data provided by Qiagen. The selected NAs percentage threshold was set to 10% and all samples passed the control of NAs and RTC. The data normalization step was performed using the endogenous miRNAs. The total execution time was 18 min and 51.02 s (Table 1) and the memory usage was 274.7 MB. Detailed instructions are available in the repository of the tool (https://bio.tools/mirkit) as well as the sample input and the output presented above.

**Table 1:** Table with the execution times in each phase

Phase	Execution time
Quality control	10.42 s
Differential analysis	2.32 s
Functional analysis	18 min 38.25 s

### Qualitative comparison to HTqPCR tool

We used HTqPCR [14], a software toolbox for dealing with RT-PCR data in order to compare its functionalities with miRkit since they have several of them in common. Also, HTqPCR is a well-known package for analyzing RT-PCR data. Both of them are written in R including quality control, normalization, clustering and differential analysis. We highlighted the clear advantages of miRkit such as the automatic process of all phases, the linking of the statistically significant miRNAs with public databases. The main differences are listed in Table 2. A clear advantage of miRkit is the automatic process of all phases. Moreover, the linking of the statistically significant miRNAs with public databases gives the user the opportunity to complete the functional analysis within the frame of miRkit.

**Table 2:** Comparison of functionalities offered by miRkit and HTqPCR

	miRkit	HTqPCR
Language	R	R
Input	Standard output from miScript miRNA PCR Array (Qiagen) technology/ Handles data from multiple plates	Data preprocessing is required/ Only single-plate data, consisting of either 96 or 384 wells
Usage	Automatic	Manual (users need to write their one code)
Quality control	Yes	Yes
Data filtering	Yes Automatically excluding samples based on NAs and on RTC	No No standard way implemented
Normalization	Yes Endogenous/exogenous genes	Yes Scaling up the values or changing the total distribution of values
Clustering	Yes	Yes
Differential analysis	Yes	Yes
Link miRNAs with databases	YES (mirtarbase, tarbase, diana_microt, etc.)	No
Enrichment analysis of deregulated genes	Yes (KEGG and GO)	No

Overall, miRkit implementation supports a fast execution with low memory usage in order to (i) perform quality control of the samples and data normalization, (ii) identify significant differences on the expression profiles of miRNAs, and (iii) link the significant miRNAs with the targeted genes and biological processes. In each step of the process, the tool produces also the relevant visual representations of the results.

Compared to the traditional commercial software for analyzing RT-PCR data, miRkit aims to become fully aligned to the FAIR principles (Findable, Accessible, Interoperable, Reusable) for Research Software [15]. With the exception of open source and a free to use tool, miRkit has the distinct advantage in terms of usage, such as the completely automated process, the data filtering based on the data quality, the linking of the differentially expressed miRNAs to genes through different databases, and the GO/KEGG enrichment analysis of deregulated genes. Finally, this package has fast execution time and uses very low memory.

Although the miRkit is focusing on one platform technology, there are many examples of commercial array platforms for which researchers developed tools in R in order to analyze their data with open source tools that are more automated and provide a better workflow for complete analysis. Using the array of 450 K Illumina as an example, there are plenty of options in R such as minfi, RnBeads, shinyMethyl, etc. Having alternative options to analyze the data, especially through workflows supporting completely automated processes from raw data to the association with genes and pathways like the proposed tool, is a critical element for the scientific community.

It is freely available on GitHub and is accompanied by detailed documentation and examples, in order to facilitate the reproducibility of the presented results. Our method provides a new perspective towards analyzing RT-PCR data. Also, it supports efficient data discovery using the gold standard approach of limma analysis and linking the information with publicly available databases to extract the biological meaning.

## Limitations

miRkit focuses on the data from miScript miRNA PCR Array (Qiagen) technology. Other miRNAs technologies which produce count tables i.e. samples on the columns and miRNA of interest on the row, could be possibly analyzed using our tool by following the format of our input files. To this end, a list of conversion scripts will be developed so that a user can use them to convert outputs from other PCR array technologies than Qiagen, as an input to miRkit. This limitation may be addressed in future versions of the miRkit.

## Data Availability

The tool is freely available under a ΜΙΤ license from https://bio.tools/mirkit, and offers fast execution times with low memory usage. The miRkit was applied on artificial data provided by Qiagen.

## Abbreviations

miRNA: MicroRNAs
RT-PCR: Reverse transcription polymerase chain reaction
miRNome: Human miRNA genome
Ct: Threshold cycle
RTC: Reverse transcription control
PPC: Positive PCR controls
NA: Not available value
GO: Gene ontology
FAIR: Findable, Accessible, Interoperable, Reusable
